# The FOOTLOOSE App: Evaluation of a Gamified App-Based Exercise Intervention for Children and Adolescents with Congenital Heart Disease—A Mixed-Methods Feasibility Study

**DOI:** 10.3390/jcdd13050199

**Published:** 2026-05-07

**Authors:** Charlotte Schöneburg, Isabel Uphoff, Anna Thußbas, Laura Willinger, Renate Oberhoffer, Peter Ewert, Jan Müller

**Affiliations:** 1 Institute of Preventive Pediatrics, Technische Universität München, 80809 München, Germany; 2 Department of Congenital Heart Disease and Pediatric Cardiology, Deutsches Herzzentrum München, Technische Universität München, 80636 München, Germany

**Keywords:** congenital heart disease, gamification, pediatric cardiology, health promotion, mobile health

## Abstract

Background: A physically active lifestyle is crucial for long-term cardiovascular health; however, access to supervised exercise programs for children and adolescents with congenital heart disease (CHD) remains limited. Although prior digital exercise interventions for this population have demonstrated safety and feasibility, adherence has often been low. Mobile health approaches integrating gamification may enhance motivation and engagement, particularly among young “digital natives.” FOOTLOOSE is an app-based home exercise program developed specifically for children and adolescents with CHD. This study aimed to evaluate user experience, usability, and perceived impact using a multimethod approach. Methods: Children and adolescents aged 10–18 years with simple, moderate, or complex CHD were recruited between July and December 2025 mainly during routine outpatient visits at the TUM Klinikum Deutsches Herzzentrum. Participants used the FOOTLOOSE app in their daily lives over a two-week period. Evaluation included semi-structured qualitative interviews and standardized questionnaires assessing physical activity self-efficacy, enjoyment of physical activity (PACES-S), user experience (UEQ), and health-related quality of life (KINDL^®^). Interviews were conducted digitally, transcribed verbatim, and analyzed using qualitative content analysis according to Kuckartz until thematic saturation was reached. Results: A total of 22 participants (mean age 13.4 ± 2.3 years; 54.5% female) were included. Overall, the FOOTLOOSE app was perceived positively, with participants highlighting enjoyment, intuitive usability, and personalized workout creation. Participants contributed diverse and creative suggestions for further app development, particularly regarding more advanced gamification features (e.g., games or rankings). Most participants reported self-perceived increase in physical activity during the intervention period (*n* = 15). UEQ scores (mean ± SD) were as follows: attractiveness (1.3 ± 0.8), perspicuity (1.7 ± 1.1), efficiency (1.2 ± 0.9), dependability (1.4 ± 0.7), stimulation (1.0 ± 1.1), and novelty (0.6 ± 1.0). Conclusions: This study demonstrates the feasibility and user acceptance of a gamified, app-based home exercise program for children and adolescents with CHD. User-centered feedback highlights important directions for iterative refinement, particularly regarding age-appropriate and engaging gamification elements. These findings provide a foundation for future studies evaluating long-term engagement and effectiveness in larger samples.

## 1. Introduction

Regular physical activity (PA) is crucial for healthy physical, psychological, and social development and contributes to the prevention of cardiovascular and metabolic diseases later in life [1,2]. The World Health Organization (WHO) therefore recommends that children and adolescents aged 5 to 17 accumulate at least 60 min of moderate-to-vigorous PA daily [3]. However, many young people fail to meet these recommendations, with activity levels being particularly low among those with chronic health conditions [4,5,6]. This is especially critical as PA habits are established early in life and often persist into adulthood [7,8]. Encouraging children and adolescents with congenital heart disease (CHD) to be physically active and providing them with structured guidance that enables them to perform exercises independently represents an important component of long-term follow-up care in this patient group.

To date, exercise interventions for children and adolescents with CHD are predominantly offered as supervised programs, which are typically available only in large metropolitan areas such as specialized cardiac centers [9,10]. Consequently, many patients have limited access to such interventions due to travel distance and organizational barriers. For this reason, there is a need to deliver exercise programs directly into patients’ home environments and to integrate them into their daily lives. A smartphone-based application appears to be a particularly suitable approach to reach children and adolescents in their everyday routines. Previous research has demonstrated the potential of mobile health (mHealth) interventions to increase PA in children and adolescents [11]. In particular, studies indicate that the use of gamification in health-related applications can positively influence behavior in this target group [12,13,14]. By incorporating playful elements, gamification can support children and adolescents in achieving recommended activity goals [15].

Gamified interventions operate through several psychological mechanisms: they can make goals transparent, motivate long-term engagement, provide immediate feedback, offer positive reinforcement, and simplify complex content. Moreover, gamification facilitates social comparison and interaction among users, thereby promoting peer support and collaborative goal pursuit. These mechanisms are consistent with established theories of behavior change, which form the theoretical basis of the present work and aim to promote a more physically active lifestyle among young patients [16].

Based on these considerations, an app-based exercise program for children and adolescents with CHD was developed and is evaluated in the present study. The exercise program had previously been implemented via a digital platform and was comprehensively evaluated in a randomized controlled intervention study [17]. The results indicated that participation led to improvements in motor performance in a subgroup of children and demonstrated that structured exercise interventions are safe and feasible for patients with CHD. However, adherence was limited, with participants completing on average only 33% of the planned training sessions [17].

To improve compliance and usability, the program was subsequently redesigned as a smartphone application (FOOTLOOSE) intended to be more modern and user-friendly than the previous platform. The application also enables a larger number of participants to be reached, independent of geographic location. This study primarily aims to evaluate the user experience, usability, and perceived impact of the FOOTLOOSE app in a real-world setting and, where appropriate, to identify user-centered directions for further improvement. Accordingly, the study was designed as an exploratory app evaluation rather than an assessment of objective effects on physical performance.

## 2. Methods

### 2.1. Study Design

The FOOTLOOSE evaluation was conducted as a mixed-methods study. This multimethod approach combines the advantages of qualitative and quantitative methods, providing a more comprehensive understanding of the suitability of user-centered interventions from different perspectives [18]. The chosen study design is characterized by a participatory approach. The literature emphasizes that continuous user experience and the involvement of the target group in the development of mHealth applications are recommended. The use of individual or group interviews is highlighted as a frequently used evaluation method, particularly well suited to generating in-depth insights into needs and expectations [19].

After two weeks, the FOOTLOOSE app (Version: 1.0.1) was evaluated qualitatively in semi-structured individual interviews and quantitatively using standardized questionnaires.

The questionnaires included user experience, enjoyment of physical activity, and activity-related self-efficacy. The study was conducted in accordance with the Declaration of Helsinki and Good Clinical Practice guidelines. Ethical approval was obtained from the Technical University of Munich (2025-211-S-SB).

### 2.2. Recruitment and Study Sample

After obtaining informed consent from participants and their legal guardians, a total of 22 children and adolescents aged 10–18 years participated in the study over a two-week period. Participants were recruited between July and December 2025 at the TUM Klinikum Deutsches Herzzentrum (*n* = 19), where they were approached on site. Additional participants were recruited via kidsTUMove at the TUM School of Medicine and Health (*n* = 3). Following recruitment, participants received on-site support from the study team for the installation of the app and an introduction to its functions.

Individuals with cognitive impairments or oncological diseases were excluded. Participation was also restricted to individuals with valid sports medical clearance and access to an iOS device. The mean age of the participants was 13.4 ± 2.3 years. The sample comprised 12 girls (54.5%) and 10 boys (45.5%). Regarding CHD severity, 11 participants were classified as having a complex heart defect, 5 as moderate, and 2 as simple, according to the American College of Cardiology/American Heart Association Task Force on Clinical Practice Guidelines [20]. In addition, two participants were diagnosed with cardiomyopathy or a cardiac arrhythmia. An overview of the sociodemographic and study-specific characteristics is presented in Table 1.

### 2.3. FOOTLOOSE Intervention

The FOOTLOOSE app can be downloaded for free in the iOS AppStore (Apple Inc., Cupertino, CA, USA). It is based on a gamification system. Users can earn virtual coins by completing training sessions and then spend them in the in-app shop to purchase items. These can then be used to design a virtual space and their own avatar.

**Figure 1:**
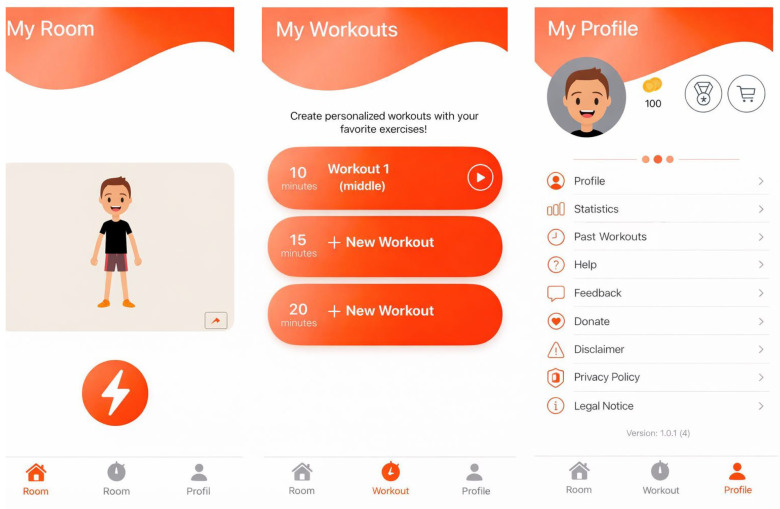
Overview of the three main sections of the FOOTLOOSE app: My Space (**left**), My Trainings (**center**), My Profile (**right**).

Participants used FOOTLOOSE for two weeks and were required to complete regular training sessions during this intervention phase. Training can take place with the FOOTLOOSE app at home or at any other location. The app comprises three main areas, which are briefly described below and visualized in Figure 1.

**Figure 2:**
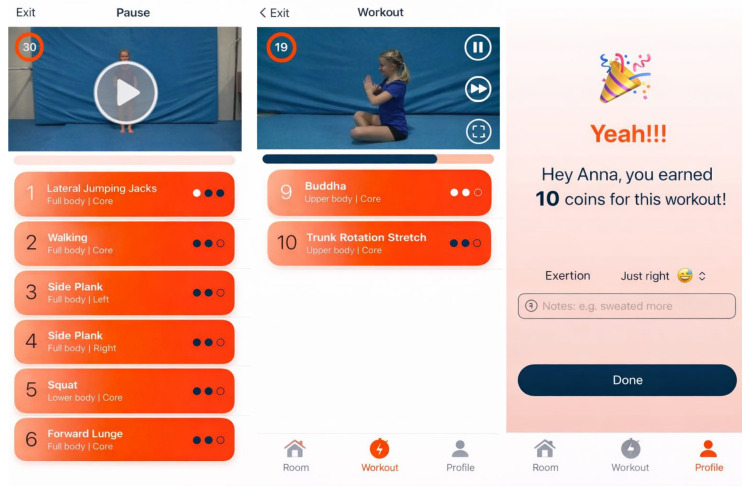
Overview of the training sessions of the FOOTLOOSE app: Workout with all exercises (**left**), Cool Down (**center**), display of earned coins (**right**).

**Figure 3:**
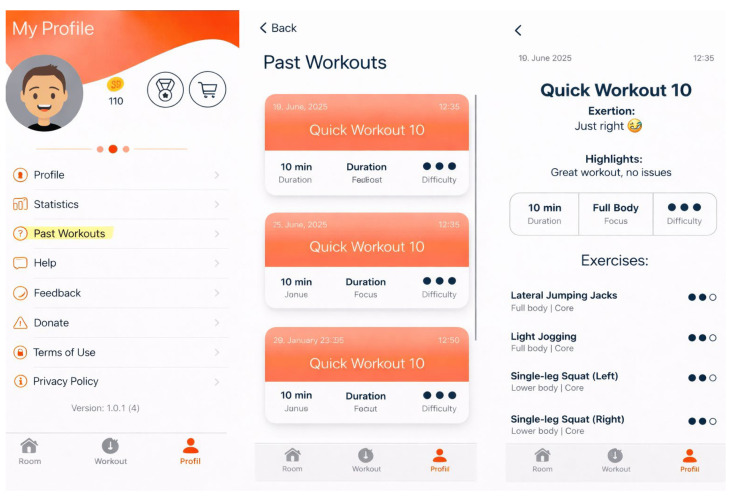
User interface of the FOOTLOOSE app: profile overview, past workouts, and detailed workout summary.

**Figure 4:**
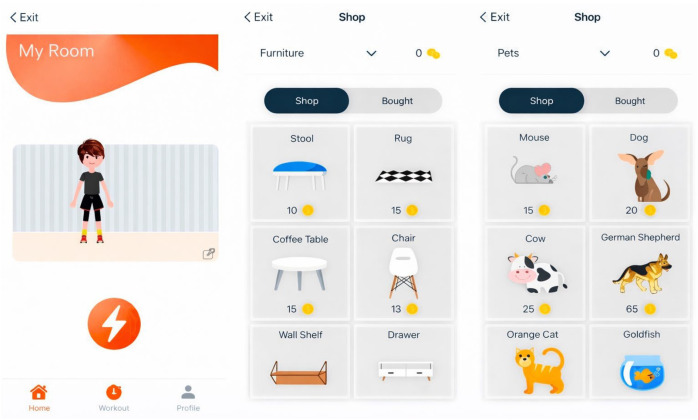
FOOTLOOSE avatar and virtual shop.

In the My Space section, participants can customize their virtual room, which is complemented by a personalized avatar. They can also start a training session with pre-selected exercises and choose the desired duration. In the My Trainings section, users can create their own training sessions by selecting exercises and defining the training time. A visualization of the training is demonstrated in Figure 2. In the My Profile section, the current balance of virtual coins earned through training is displayed. Items (e.g., plants, animals, furniture) that can be purchased in the integrated app shop can be exchanged for coins and placed in the virtual room. Furthermore, settings and overviews of completed training sessions and statistics are stored there; see Figure 3 and Figure 4.

Training sessions are guided by short, child-friendly videos. The sessions cover content from the areas of strength, flexibility, coordination and relaxation (e.g., arm circles, trunk bends, mountain climbers, forearm support). Each training session is similar in terms of its structure: Typically, they begin with a short warm-up, followed by the main part. The final part consists of a cool-down and short stretching exercises. The duration of the sessions can be set to 10, 15 or 20 min as required. 30 s are planned for each exercise and rest period. After completing the training, the number of earned coins is displayed. As an optional tool, participants can add notes and information about the intensity.

### 2.4. Qualitative Assessment

After two weeks of using the FOOTLOOSE app, semi-structured interviews were conducted via Zoom Communication, Inc. (Version: 6.7.7; San Jose, CA, USA). A pre-prepared interview guide containing open questions was used for this purpose (Table 2).

An introductory question designed to create a trusting conversation atmosphere was included. After that, the general attitudes towards smartphone use, health apps and physical activity were queried.

The main part of the interview focused on the participants’ experiences and evaluations of the FOOTLOOSE app. This included aspects such as the user experience, activity promotion and sustainability. At the end of the interview, the participants were given the opportunity to ask questions and make comments. Recruitment was guided by the aim of achieving thematic saturation in the qualitative interviews [21].

### 2.5. Quantitative Assessment

#### 2.5.1. Physical Activity Self-Efficacy

Activity-related self-efficacy in youth describes the belief in one’s capability to participate in PA and to overcome barriers to engagement [22]. Evidence from multiple systematic reviews highlights it as a consistent psychological correlate and key predictor of PA behavior in children and adolescents [23,24,25]. Physical activity self-efficacy was assessed using the validated German scale for self-efficacy in relation to PA [26,27]. The instrument comprises six items on self-efficacy and two items on social support from family and friends. Responses are given on a five-point Likert scale (1 = “agree not at all” to 5 = “agree completely”), with higher scores indicating stronger self-efficacy.

#### 2.5.2. Enjoyment of Physical Activity

Enjoyment of physical activity was assessed using the short version of the PACES-S [28]. This instrument is often used in intervention studies, as PA enjoyment is associated with the implementation and adherence to activity recommendations [29]. For children and young people, there are indications of reliability and validity for the German version [30]. The scale comprises four statements (“It makes me happy”, “I find it pleasant”, “It is very pleasant”, “It feels good”), which are answered on a 5-point Likert scale from 1 (“I strongly disagree”) to 5 (“I fully agree”). Higher values indicate greater enjoyment of physical activity.

#### 2.5.3. User Experience

The participants’ user experience was assessed using the user experience questionnaire (UEQ) [31]. It comprises 26 items and covers six quality dimensions of interactive products. These are attractiveness, clarity, efficiency, reliability, stimulation and novelty. The items are formulated as bipolar adjective pairs and are answered by the participants on a 7-point scale. The polarity is arranged in a different order for each item. The values were transformed using the official UEQ Excel Data Analysis Tool [32], with values ranging from −3 to +3. Values between −0.8 and 0.8 are considered neutral, values above 0.8 are considered positive, and values below −0.8 are considered negative. Good reliability and adequate construct validity are reported for the UEQ [33].

#### 2.5.4. Health-Related Quality of Life

In the quantitative analysis of health-related quality of life (HRQoL) in children and young people, the KINDL^®^ was used as the measurement instrument in the study. Depending on their age, two different versions were used: Kid-KINDL for participants aged 7–13, and Kiddo-KINDL for 14–17 year olds. The 24 items cover six dimensions (physical and mental well-being, friendships, self-esteem, family relationships, and coping with everyday life). Each of these sub-scales contains four items, with higher item values generally indicating a better HRQoL. However, some questions are formulated in the opposite direction, meaning that high values indicate a lower HRQoL. These items were accordingly recoded. For the evaluation, the mean values were first formed within each subscale and then multiplied by the respective item number to obtain a scale sum value [34]. For the analysis, scale and total values were calculated from the respective item values and then converted to transformed values from 0 to 100. The German KINDL^®^ is considered a reliable, valid and practical instrument for measuring HRQoL in children and adolescents [34].

### 2.6. Data Analysis

The data from the interviews on the perception and use of the FOOTLOOSE app were evaluated using qualitative content analysis according to Kuckartz [35]. After verbatim transcription, categories were developed in combination with a deductive-inductive approach. The starting point was the research question and the derived top categories, which were refined over time. Relevant text passages were assigned to the categories, thereby forming codes. The coding was carried out by the person conducting the interview. The software MAXQDA (Version: 26.2; VERBI Software GmbH; 24th ed.; Berlin, Germany) was used for data organization and coding management.

Quantitative data were analyzed as follows: Group differences were evaluated with an independent-samples *t*-test for normally distributed data and the Mann–Whitney U test for non-normally distributed data.

## 3. Results

### 3.1. Qualitative Results

The interviews initially covered three broad themes: smartphone use, health apps and PA, to understand the participants’ views on these aspects. The interview data were analyzed using a qualitative content analysis according to Kuckartz [35]. These included the general impression, perceived strengths and weaknesses, assessment of the gamification elements, ideas for improvement and the impact of FOOTLOOSE on physical activity.

### 3.2. Smartphone Usage

Of the 22 children and young people, 18 had their own smartphone (10 girls, 8 boys), while four participants did not have their own device. The most common daily usage time was 2–3 h (*n* = 6). Overall, reported usage times were predominantly between 1 and 4 h per day. At the lower end, one boy reported using his smartphone for only a few minutes per day, and one boy reported a daily usage time of up to 30 min. At the upper end, one boy reported using his smartphone for 5–6 h per day. Two girls reported that their daily smartphone use was limited to one hour. Smartphones were used primarily for messaging and entertainment purposes, such as watching videos (e.g., YouTube), using social media, and playing games.

### 3.3. Perception of Health Apps

Many of the children and young people surveyed associated health apps primarily with general health issues (*n* = 5; 4 girls, 1 boy) and healthy eating (*n* = 10; 6 girls, 4 boys). These types of applications were seen by the participants as a way to support and improve their own health. Furthermore, health apps were often described as helpful tools for building fitness, stamina and endurance, and were therefore directly associated with sports and training. Some participants also mentioned the connection to smartwatches, which can record specific values such as step count, sleep or heart rate. Two participants reported using a smartwatch, while others used a pedometer app. As well as these associations, a running app was mentioned as an example. One boy, who had no prior experience with health apps, imagined such an app as a reminder function that encourages you to put your phone down and go outside.

### 3.4. Attitude to Physical Activity

All participants except one reported being regularly physically active and exercising (*n* = 21). The only participant who did not engage in recreational sports was one girl; however, she still considered physical activity worthwhile and reported feeling comfortable during school sports. Overall, the study population experiences PA as something positive and associates it with joy and increased well-being. However, some respondents also mentioned fatigue or physical discomfort, such as side stitches or shortness of breath. Furthermore, some children and young people described sport as a psychological outlet.

### 3.5. Overall Impression

All participants rated FOOTLOOSE as a positive experience, reporting enjoyment during use and indicating a desire to continue using the app. This was also directly expressed in the interviews, for example, with statements such as “It was great fun” (girl, 14 years) or “I really liked the app” (boy, 10 years). In particular, the children emphasized that they could create their own training plan (boy, 15 years). One girl (10 years) found FOOTLOOSE motivating for more movement. However, it was also suggested that the app might be less appealing to older users. The app was used regularly overall. Most children and teenagers used it almost daily or every other day, while the least frequent usage was once a week. All available training durations were used, with 10 and 20 min sessions being the most popular. The reasons given by the participants for their intention to continue using the app were primarily the fun of training (boy, 15 years), the practical possibility of consulting suitable sports from home (girl, 13 years) and the general interest in being active (girl, 10 years). FOOTLOOSE was also used to occupy oneself during periods of boredom (boys, 15 and 16 years).

### 3.6. Strengths and Weaknesses

The ability to create individual workouts or a personal training plan was identified as a key strength of FOOTLOOSE (“I liked that I could create my own training plan” (girl, 15 years)). Some participants emphasized the flexibility to select exercises according to personal preferences. A 16-year-old girl said that she “always did exercises that she enjoyed”. Others liked the training because they learned new exercises through the app. It was also positively highlighted that the duration of the training sessions can be set independently, which was perceived as practical by the users. This allowed them to adapt the training to their available time and motivation. Further positive aspects were the “intuitive” design and simple handling (boy, 15 years). A 12-year-old boy said, “It’s really easy to use”. Furthermore, the ability to use the app anywhere (boy, 12 years) and motivating elements such as coins (girl, 10 years) and the shop were described as positive. Some also reported that the app was used by siblings (girl, 12 years) and as an alternative on training-free days (boy, 12 years) or when there was little time (girl, 14 years).

As weaknesses, some of the teenagers said that FOOTLOOSE was not sufficiently age-appropriate (1 boy, 3 girls, 14–17 years). Furthermore, the pause function was criticized multiple times (2 girls, 2 boys). The “Pause” display was too small and was therefore easily overlooked. Furthermore, some participants found it “a bit silly” that the break was so long (girl, 13 years). Furthermore, technical and practical access barriers were described by the participants. The app was only usable on iOS devices, and the installation was perceived as time-consuming. As not all respondents had their own Apple device, they had to use other people’s devices (e.g., family members), which could restrict usage frequency. Some participants also reported that they found some of the exercises too easy.

### 3.7. Perception of Gamification Elements

The shop and the virtual room were evaluated inconsistently by the children and young people: Some found these elements an enjoyable change (boy, 12 years), while others perceived the offer as limited (“You can’t really do much with the items, except put them in the room”) and complained that you could only buy things for the beach (girl, 12 years). The coins were described as motivating but were also seen as more of an incentive for younger users (girl, 16 years). Additionally, some participants were initially unaware that the shop, room, and coin system existed or how they were used.

### 3.8. Ideas for Improvement

The most important suggestions for improvement were an extension of the shop, particularly with regard to more items and backgrounds (5 girls, 2 boys). Furthermore, they suggested more interesting and engaging gamification elements to make the app more varied. A 14-year-old girl said, “Maybe change something so it’s a bit more interesting”. Some participants referred to the “Anton App” (solocode GmbH, Berlin, Germany) or “Duolingo” (Duolingo, Inc., Pittsburgh, PA, USA) as examples, as these also use playful elements. Concrete ideas included rankings, trophies or additional games.

Further suggestions for improvement related to functional aspects: One participant suggested that FOOTLOOSE should integrate a notification system to remind him to train. It was also suggested that breaks within the workouts could be made more obvious. It was also suggested that an audio function could explain the next exercise and provide additional information on how to perform it correctly during the breaks. However, many children and young people (*n* = 16) also reported that they are generally satisfied with FOOTLOOSE and do not consider any further changes to be necessary.

### 3.9. Impact of FOOTLOOSE on PA

The majority of participants (*n* = 15, 7 girls, 8 boys) reported a perceived increase in their PA or had at least noticed a slight increase in their activity due to using FOOTLOOSE. The app was primarily described as a motivating factor in integrating movement more frequently into daily life. A 15-year-old boy exemplified this, saying, “Now I do it almost every day, in the evening, just a little. Before, I wouldn’t have done it”. A girl also said that she did the exercises while doing other activities, such as watching TV, and that this reduced her sitting time. However, other participants did not notice any change in their PA, often citing that they had already been active before. A 10-year-old boy expressed the following opinion on this matter: “No, not really, because I’m active a lot anyway”.

### 3.10. Quantitative Results

The questionnaire results are presented in Table 3 as mean ± standard deviation. Overall, no significant gender differences were observed for the UEQ scales (Attractiveness: 1.3 ± 0.8; Perspicuity: 1.7 ± 1.1; Efficiency: 1.2 ± 0.9; Dependability: 1.4 ± 0.7; Stimulation: 1.0 ± 1.1; Novelty: 0.6 ± 1.0), PACES-S (4.2 ± 0.7) and GPA (3.5 ± 0.8). Significant differences were found exclusively in the KINDL (HrQoL) subscales physical well-being (*p* = 0.019) and school (*p* = 0.003).

## 4. Discussion

The present mixed-methods evaluation investigated the acceptance, usability, and perceived impact of the app-based training intervention FOOTLOOSE. Thus, to our knowledge, this app represents the first of its kind to offer a disease-specific, home-based training program for children and adolescents with CHD. Overall, the findings indicate good acceptance of the intervention within the target group. Participants described the application as enjoyable, practical, understandable, intuitive and easy to use. In addition, participants perceived the app as supportive for integrating more physical activity into their daily lives. At the same time, the results identified specific areas for improvement, particularly regarding motivational features and content variety.

A central finding of this study was the favorable evaluation of the personalized training structure. Participants appreciated the possibility to adapt exercises in the app to their individual capabilities and daily conditions. This observation aligns with previous literature indicating that interventions incorporating personalized training content or individual exercise prescriptions could achieve higher adherence and improvements in activity behavior [36,37,38]. However, such effects were not assessed in the present study. Digital interventions seem particularly effective when they offer customizable features and motivational support through age-appropriate activities [36,37,38,39,40]. Personalization may therefore represent an important mechanism supporting engagement and sustained use in home-based exercise programs for children and adolescents with CHD.

In addition, gamification emerged as a key component influencing user experience. The majority of participants responded positively to the playful elements of FOOTLOOSE such as collecting coins. Previous gamified mobile applications to improve PA have shown that gamification elements can provide motivational support and facilitate goal attainment [41,42]. For example, gamification elements such as leaderboards, points, and integrated badges have been shown to support schoolchildren in achieving their daily PA goals [41], while building blocks on a smartphone application in public playgrounds encourage children to remain physically active in an outdoor setting [42]. In addition, MedBIKE integrates both real-time monitoring on a bicycle ergometer and interactive gaming elements by combining live-streamed exercise sessions with game-based activities during workouts. This approach was found to enhance adherence and was perceived by Fontan patients as an enjoyable form of exercise [37,43].

However, age-related differences became apparent. Younger children appeared to be more motivated by reward systems and collection mechanics, whereas older adolescents partly perceived these elements as less motivating. This suggests that the effectiveness of gamification depends less on isolated features and more on an age-appropriate and coherent overall design [44]. Effective gamification should therefore promote autonomy, provide orientation through feedback, and match task difficulty to individual abilities [16,44]. As concrete suggestions for improvement, the participants named more shop items and items with clear functions to promote motivation and make the app more interesting. Furthermore, it was shown that ‘challenges’ or task-based goals can be perceived as a useful addition to strengthen motivation beyond mere reward. Such challenges could support the principle of gamification by making clear goals, feedback and progress more visible and increasing engagement [16]. For children and adolescents with CHD, however, flexibility remains essential, as performance capacity can vary considerably between individuals and across health conditions [10,45]. Future developments should therefore allow adjustable difficulty levels and optional challenges to ensure both safety and sustained motivation. Consistent with this, the UEQ subscale “Novelty” was rated lower, suggesting that the app’s content variety and design may have been less engaging, particularly for older adolescents.

Engagement also appeared to depend on individual user characteristics. Differences in acceptance were not only related to age but also to pre-existing interests and activity-related attitudes. Prior research indicates that perceived usefulness, social influences, and user attitudes are important predictors of technology acceptance [46]. In particular, familiarity with digital games and positive attitudes toward PA may influence how users perceive and interact with a gamified health application. It should be noted that most participants reported already enjoying physical activity and engaging in sports prior to study participation. Consequently, app-based interventions may appeal more strongly to certain subgroups within the target population. Providing optional motivational approaches, such as playful rewards, progress tracking, or structured guidance, may therefore help accommodate heterogeneous user preferences.

Another relevant aspect concerned social features. Several participants requested opportunities for social interaction or competition. Social gamification has been shown to facilitate peer networking and mutual support [16], and optional functions such as team participation or peer interaction could therefore contribute to longer-term adherence [41]. Similarly, integrating self-monitoring and feedback may further support continued usage, as regular monitoring in mHealth applications has been associated with sustained engagement [47]. In this context, it is noteworthy that FOOTLOOSE already incorporates self-monitoring features, such as tracking training frequency and allowing users to reflect on perceived exertion after each session. These features may further support engagement by enabling users to monitor their progress and reflect on their activity behavior. Future developments could further enhance these functions by integrating wearable devices such as smartwatches, enabling objective tracking of activity levels and providing real-time feedback to users.

While some adjustments are still needed to increase long-lasting incentives, behavior change toward a permanently active lifestyle is complex and rarely achieved through a single intervention alone [48]. Behavioral interventions aiming to increase PA frequently encounter difficulties in maintaining long-term adherence [49]. Nevertheless, digital applications offer the opportunity to deliver prevention and health promotion in a modern and age-appropriate format. The present findings therefore suggest that app-based interventions such as FOOTLOOSE may serve as a valuable complement to conventional follow-up care for pediatric CHD patients. From a clinical perspective, the FOOTLOOSE app may represent a low-threshold complement to conventional follow-up care in pediatric cardiology, particularly for patients with limited access to supervised exercise programs. By integrating structured, home-based activity into daily life, such approaches may help support long-term engagement with PA in this population.

### Limitations

Several limitations should be considered when interpreting the results. First, the sample size was small (*n* = 22), which may limit generalizability. In addition to that, the study population was clinically heterogeneous, including patients with varying CHD severity as well as cardiomyopathy and arrhythmia, which may differ in exercise capacity and risk profile. Due to the small sample size, subgroup analyses were not feasible. Second, the usage period was relatively short (two weeks), meaning that the results primarily reflect initial user experiences rather than sustained behavior change, long-term adherence, or intervention effectiveness. The positive responses may partly reflect novelty effects. Third, qualitative interviews may be affected by socially desirable responding [50]. In addition, the evaluation relied largely on subjective self-reports, preventing any conclusions regarding objective changes in PA or functional improvement. Thus, the present study should be interpreted as an evaluation of user experience and perceived impact rather than an effectiveness study. Also, the questionnaires were only measured once, post-intervention, and thus cannot provide information on the impact of the FOOTLOOSE intervention. Lastly, the app was only available for iOS devices, which may have introduced accessibility and socioeconomic bias.

## 5. Conclusions

In summary, the FOOTLOOSE app was well accepted by children and adolescents with CHD and was perceived as an enjoyable, practical, and understandable tool that may support perceived PA in everyday life from the users’ perspective. The findings provide specific directions for further app development, particularly regarding additional content, enhanced motivational elements, and expanded personalization options. Beyond optimizing the application itself, the findings provide broader insights into the design of digital interventions aimed at promoting perceived PA in pediatric patients with chronic conditions and highlight their potential not only as a substitute but as a complement to traditional exercise programs in pediatric cardiology.

## Figures and Tables

**Table 1:** Study population characteristics (*n* = 22).

Characteristics		
Age (*n* = 22) (years; mean ± SD)	13.4 ± 2.3	
Sex (*N* = 22)	*n*	Proportion/%
Female	12	54.5
Male	10	45.5
CHD severity (*N* = 22) *	*n*	Proportion/%
Simple	2	9.1
Moderate	5	22.7
Complex	11	50.0
Cardiomyopathy	2	9.1
Cardiac Arrhythmia	2	9.1

* based on the American College of Cardiology/American Heart Association Task Force on Clinical Practice Guidelines [20].

**Table 2:** Semi-structured interview guide.

Section	Outcome	Key Question	Follow-Up Question
Introduction	General attitude towards smartphone use	What do you normally use your smartphone for?	Roughly how much time do you spend on your smartphone daily?
	General attitude towards digital health apps	What do you think a health app is?	Which health apps do you know? What do you think they are for?
	General attitude towards physical activity	What is your attitude towards sports and physical activity?	Do you have any sports hobbies? Which ones? How do you feel when doing sports?
Main Part	Evaluation of the FOOTLOOSE app	How did you like the FOOTLOOSE app?	What did you particularly like? Which video or exercise did you enjoy the most? What did you not like? What would you change about the app? Were you satisfied with your virtual room and the offers in the app shop?
	Long-term use/sustainability	Can you imagine using the FOOTLOOSE app long-term?	Which time unit did you like best? (10 min, 15 min, 20 min) How many minutes per week did you train with the app? Or: How often per week did you use the app? Would you like to continue using the app? Why? What would have to change so you would keep using it?
	Activity promotion through FOOTLOOSE app	Has the FOOTLOOSE app influenced your activity behaviour?	In what way did the app motivate you to train more? What do you think about doing extra training sessions to collect more coins for your virtual room?
Closing	Closing remarks by interviewer	Do you have any further questions or comments about the app?	Is there anything else you would like to say or ask?

**Table 3:** Results of the questionnaires separated by gender (mean ± standard deviation).

Questionnaires	All (*n* = 18)	Boys (*n* = 6)	Girls (*n* = 12)	*p*-Value *
**UEQ**				
Attractiveness	1.3 ± 0.8	1.1 ± 0.9	1.4 ± 0.7	0.533
Perspicuity	1.7 ± 1.1	1.4 ± 1.2	1.8 ± 1.0	0.554
Efficiency	1.2 ± 0.9	1.1 ± 1.0	1.2 ± 0.8	0.932
Dependability	1.4 ± 0.7	1.2 ± 0.9	1.6 ± 0.6	0.404
Stimulation	1.0 ± 1.1	1.0 ± 1.2	1.0 ± 1.1	0.962 ^
Novelty	0.6 ± 1.0	0.9 ± 0.9	0.4 ± 1.1	0.672 ^
**PACES-S**	4.2 ± 0.7	4.3 ± 0.8	4.2 ± 0.7	0.701 ^
**GPA**	All (*n* = 19)	Boys (*n* = 7)	Girls (*n* = 12)	
	3.5 ± 0.8	4.0 ± 0.9	3.3 ± 0.8	0.111
**KINDL**	All (*n* = 18)	Boys (*n* = 7)	Girls (*n* = 11)	
Total	76.6 ± 8.9	81.4 ± 8.3	73.5 ± 8.0	0.068
Physical well-being	71.5 ± 18.5	83.0 ± 12.4	64.2 ± 18.4	**0.019**
Psychological well-being	84.1 ± 12.2	82.1 ± 15.1	85.3 ± 10.6	0.644
Self-worth	65.3 ± 17.3	67.9 ± 17.5	63.6 ± 17.9	0.628
Family	88.2 ± 9.8	89.3 ± 7.8	87.5 ± 11.2	0.969
Friends	77.1 ± 15.0	82.1 ± 11.1	73.9 ± 16.7	0.225
School	73.3 ± 12.2	83.9 ± 4.9	66.5 ± 12.3	**0.003 ^**

* *p*-values were calculated using a *t*-test for independent samples with significance *p* ≤ 0.05; the Mann–Whitney U-test was used for non-normal distributed data (^). Normal distribution was tested using the Shapiro–Wilk test. User Experience Questionnaire (UEQ): Scale −3 to +3 (values < −0.8 = negative evaluation, >+0.8 = positive evaluation). Physical Activity Enjoyment Scale (PACES-S) and the German scale for self-efficacy in relation to physical activity (GPA): scale 0–5 (higher values = better), health-related quality of life—KINDL (transformed scale): scale 0–100 (higher values = better).

## Data Availability

The raw data supporting the conclusions of this article will be made available by the authors on request.

## Abbreviations

The following abbreviations are used in this manuscript:

WHO	Physical Activity Health Literacy Scale for Children
PA	Physical Activity
PACE-S	Physical Activity Enjoyment Scale (Short Version)
UEQ	User Experience Questionnaire
KINDL	Health-Related Quality of Life Questionnaire
HRQoL	Health-related quality of life
GPA	German physical activity self-efficacy scale
